# A Simple Step Test to Estimate Cardio-Respiratory Fitness Levels of Rheumatoid Arthritis Patients in a Clinical Setting

**DOI:** 10.1155/2013/174541

**Published:** 2013-12-23

**Authors:** Jennifer K. Cooney, Jonathan P. Moore, Yasmeen A. Ahmad, Jeremy G. Jones, Andrew B. Lemmey, Francesco Casanova, Peter J. Maddison, Jeanette M. Thom

**Affiliations:** ^1^School of Sport, Health and Exercise Sciences, Bangor University, George Building, Bangor, Gwynedd LL57 2PZ, UK; ^2^School of Medical Sciences, Bangor University, Brigantia Building, Penrallt Road, Bangor, Gwynedd LL57 2AS, UK; ^3^Department of Rheumatology, Betsi Cadwaladr University Health Board (West), Llandudno General Hospital, Llandudno LL30 1LB, UK; ^4^Institute of Clinical and Biomedical Science, Medical School, University of Exeter, Exeter EX1 2LU, UK

## Abstract

*Purpose*. Exercise tests represent an important clinical tool to evaluate cardio-respiratory fitness and to predict future adverse cardiovascular events. However, use of such tests in patients with rheumatoid arthritis (RA) is relatively uncommon despite well-established evidence that low exercise capacity and high CVD mortality are features of this disease. Therefore, this study examined the validity and reliability of a sub-maximal step test for use in RA patients. *Methods*. Thirty patients (24 females) (mean ± SD age 53 ± 10 years) performed a sub-maximal step test on two occasions to estimate the criterion measure of cardio-respiratory fitness ($\dot{\text{V}}{\text{O}}_{2\max}$). A further maximal cycling test provided a direct fitness measurement ($\dot{\text{V}}{\text{O}}_{2\;\text{peak}}$). Pearson correlation coefficient, intraclass correlation coefficient (ICC), Bland and Altman plots, and 95% limits of agreement (LOA) were used to determine the validity and reliability of the sub-maximal test. *Results*. Estimated $\dot{\text{V}}{\text{O}}_{2\max}$ correlated well with directly measured $\dot{\text{V}}{\text{O}}_{2\;\text{peak}}$ (*r* = 0.79, LoA ±5.7 mL*·*kg^−1^ *·*min^−1^). Test-retest reproducibility for estimated $\dot{\text{V}}{\text{O}}_{2\max}$ was excellent (ICC = 0.97, LoA ±2.2 mL*·*kg^−1^ *·*min^−1^). *Conclusion*. The sub-maximal step test studied here represents a valid and reproducible method to estimate cardio-respiratory fitness in RA patients. This test may be useful for the assessment and management of CVD risk in a clinical setting.

## 1. Introduction

Physical fitness reflects the overall ability to perform activities of daily living [1]. Data from numerous epidemiological studies indicate that low cardio-respiratory fitness is a strong independent risk factor for all-cause and cardiovascular disease (CVD) mortality in asymptomatic individuals, persons with comorbid conditions (hypertension, obesity, and type 2 diabetes mellitus), and those with established coronary artery disease [2]. It is reported that the strength of association between low cardio-respiratory fitness and mortality is comparable to that between mortality and traditional CVD risk factors such as obesity, hypertension, hypercholesterolemia, and smoking [3–5]. This association is very important, especially for clinical populations known to have an exacerbated CVD risk. One such population is patients with rheumatoid arthritis (RA). It has been shown that CVD accounts for up to 50% of deaths in RA [6], and, typically, CVD events occur earlier, and to a greater extent in this population relative to age-matched healthy controls, and sometimes even before the fulfilment of all criteria of RA [7]. It is hypothesised that inflammation is the major contributor to CVD in RA [7] with other traditional risk factors having a less significant role than that observed in the general population [8]. However, RA patients have also been shown to have alarmingly low levels of physical fitness—20–30% lower in comparison to individuals without RA [9]. This is likely due to physical inactivity during inflammatory stages of the disease and continued physical inactivity during stages of disease remission. Despite cardio-respiratory fitness being widely recognised as an important health indicator, the assessment of fitness is often overlooked from a clinical perspective compared with other CVD risk factors [10].

The criterion measure of cardio-respiratory fitness is maximal oxygen uptake ($\overset{.}{\text{V}}{\text{O}}_{2\max}$) typically expressed in litres of O_2_ consumed per minute (L·min^−1^) or millilitres of O_2_ consumed per kilogram of body mass per minute (mL·kg^−1^·min^−1^). Accurate measurement of $\overset{.}{\text{V}}{\text{O}}_{2\max}$ usually requires expensive testing systems, treadmills or cycle ergometers, suitably trained personnel; and, maximal effort from the subject [11]. It could be argued, therefore, that the requisites for measuring $\overset{.}{\text{V}}{\text{O}}_{2\max}$ might preclude routine assessment of cardio-respiratory fitness in patients “at risk” or incapable of maximal exercise. On the other hand, $\overset{.}{\text{V}}{\text{O}}_{2\max}$ can be estimated relatively easily from a short bout of sub-maximal exercise lasting approximately from 3 to 9 minutes.

A wide variety of predictive sub-maximal exercise protocols are available for use; these include treadmill walking [12, 13] and cycle ergometry [14] tests. However, motorised treadmills and cycle ergometers are not always available in a clinical setting. In contrast, step tests that require limited equipment (i.e., step, metronome, heart rate monitor, and stop watch) represent an attractive modality for assessing cardio-respiratory fitness in clinic. Since the earliest reported step test, now known as the Harvard step test [15], numerous sub-maximal step test protocols have been developed. These include the Queens College step test [16], the Canadian home fitness test [17], the Chester step test [18], and the Siconolfi step test [19].

Predictive exercise tests are population specific and to our knowledge the validity and the reliability of a sub-maximal step exercise test in RA has not been established. Therefore, the purpose of this study was to determine the validity and reliability of a predictive sub-maximal step test protocol in patients with RA. The Siconolfi protocol was chosen over others as it may be completed at relatively low levels of exercise. This is very important in low active, clinical groups like patients with RA where exercise intolerance is a feature of their disease. 

## 2. Methods

### 2.1. Sample Size

To determine the sample size, an online power calculator was used [20]. Based on Cohen's values [21], a correlation of 0.5 or greater represents a strong correlation. For the current power calculation, a correlation of 0.8 was used to represent a strong correlation. Assuming a Type 1 and Type 2 error of 5% and 20%, respectively, this resulted in a sample size of 10 participants. Therefore, we aimed to recruit 30 participants in order to allow for drop outs and missing data.

### 2.2. Patients

**Figure 1:**
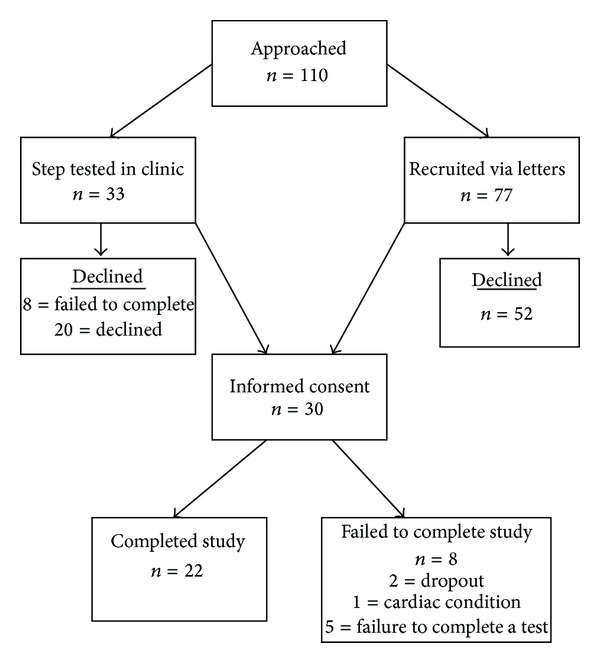
Flow chart displaying number of RA patients who were approached, recruited and completed the study.

With ethical approval, a prospective validation study was conducted in adults attending rheumatology out-patient services of the Betsi Cadwaladr University Health Board (West). Patients diagnosed with RA according to the American Rheumatism Association's 1987 Criteria for the Classification of Rheumatoid Arthritis [22], who attended the rheumatology clinic, were considered as potential participants for this study. Exclusion criteria were a current RA flare, joint surgery in the preceding two months, patients taking beta blockers, established cardiovascular disease, recent upper respiratory tract infection, and, history of substance abuse. Consequently, 30 individuals (24 females) with RA provided written informed consent and entered the study (Figure 1).

### 2.3. Protocol

Participants attended Llandudno General Hospital for testing on two occasions. The visits were separated by 1 to 3 weeks and scheduled for the same time of day. Participants were instructed to avoid performing strenuous exercise 24 hours prior to testing and not to consume any food, caffeine, alcohol, or tobacco in the 3 hours before being assessed.

#### 2.3.1. Visit One

Height and body mass were measured by standard procedures, and body mass index (BMI) was calculated (kg·m^2^). The systolic and diastolic blood pressures were taken by the standard auscultatory technique. Functional status (disability) measures were determined using the Stanford Health Assessment Questionnaire (HAQ) [23]. Disease activity was assessed using the disease activity score based on 28 joint assessments (DAS28), a validated tool for estimating RA disease [24]. Then, each participant undertook the Siconolfi step test, which has been described previously [19]. Briefly, this sub-maximal test consists of stepping up and down from a portable 10 inch (25.4 cm) step for 3 minutes per stage, for a maximum of three stages. Initially, the stepping rate for stage 1 is 17 steps per minute; if required, this is increased to 26 and 34 steps per minute for stages 2 and 3, respectively. Stepping rate is kept constant for each stage using a metronome. If the heart rate at the end of a stage, measured by telemetry (Model RS400, Polar Electro OY, Finland), is less than 65% of that predicted by the maximal heart rate prediction equation (i.e., 220—age), the participant is instructed to complete another stage. Each stage is separated by 1 minute of rest. Each individual's $\overset{.}{\text{V}}{\text{O}}_{2\max}$ is estimated from the exercise heart rate at the end of the test according to established equations [25].

Upon completion of the step test, each participant was familiarized with the equipment and procedures for a graded exercise test to volitional exhaustion using a cycle ergometer (Ergomedic 828E, Monark Exercise AB, Sweden); this included familiarization with the Borg categorical scale for rating perceived exertion (RPE) [26].

#### 2.3.2. Visit Two

During the second testing session, each participant repeated the step test. Then, after a 30-minute period, a 12-lead ECG was performed at rest. If the ECG trace was normal, participants performed a $\overset{.}{\text{V}}{\text{O}}_{2\max}$ test. This test is a direct measurement of maximal oxygen uptake (cardio-respiratory fitness). Typically, $\overset{.}{\text{V}}{\text{O}}_{2\max}$ tests can be performed on a treadmill or cycle ergometer. For this study, a maximal cycle test was chosen to determine maximal oxygen consumption rather than a treadmill protocol for several reasons. Firstly, cycle ergometer and step tests have been shown to yield similar $\overset{.}{\text{V}}{\text{O}}_{2\max}$ results, with both methods having a tendency to result in lower $\overset{.}{\text{V}}{\text{O}}_{2\max}$ values when compared to treadmill tests [27]. Secondly, maximal cycling tests in older and inactive adults increase safety by allowing smaller gradations in work and a higher quality ECG. The test involved pedalling at a constant rate of 50 revolutions per minute (rpm) for two minutes with no resistance added to the flywheel. Thereafter, resistance increased in increments of 25 watts every two minutes, until volitional exhaustion. Expired gases and air flow were monitored breath-by-breath using an automated system (800Ergo test, ZAN GmBH, Germany). Heart rate and ratings of perceived exertion (RPE) were measured at the end of every two-minute stage [26]. It was anticipated that many of these deconditioned patients would not be able to obtain a true maximal aerobic capacity, defined as a plateau in oxygen consumption during the final stage, maximal heart rate >85% of age-adjusted predicted maximal heart rate (220—age), respiratory exchange ratio (RER) > 1.10, and ratings of perceived exertion (RPE) > 17 [28]; therefore, the highest $\overset{.}{\text{V}}{\text{O}}_{2}$ recorded during the maximal cycling exercise test was considered to be the $\overset{.}{\text{V}}{\text{O}}_{2\;\text{peak}}$ value [29].

### 2.4. Data Analysis

The primary outcome measure obtained during each of the step tests was estimated $\overset{.}{\text{V}}{\text{O}}_{2\max}$ (expressed as mL·kg^−1^·min^−1^), determined using the equations developed by Siconolfi et al. [25]. Outcome measures obtained during the cycling test to volitional exhaustion included $\overset{.}{\text{V}}{\text{O}}_{2\;\text{peak}}$, peak heart rate, RER, and ratings of perceived exertion (RPE).

### 2.5. Statistical Analysis

Data was entered into a database and statistical analyses were performed (SPSS, version 19 for Windows, SPSS, Chicago, IL). The mean and standard deviation (SD) were calculated for normally distributed data. The concurrent validity of $\overset{.}{\text{V}}{\text{O}}_{2\max}$ estimated from the Siconolfi step test was assessed using the Pearson correlation coefficient (*r*) and the Bland and Altman technique [30]. The paired  *t*-test was used to establish whether there was a significant systematic bias between test measurements; a two-tailed  *P* < 0.05  was considered significant. The standard error of the estimate (SEE) was also calculated. The inter-day reproducibility of estimated $\overset{.}{\text{V}}{\text{O}}_{2\max}$ was assessed using the intraclass correlation coefficient (ICC), Pearson correlation coefficient and the Bland and Altman technique. The test-retest within-subject coefficient of variation was also calculated.

## 3. Results

One hundred and ten consecutive patients were contacted over 13 months regarding potential participation in the study. Of these, 80 were unwilling to participate; thus, 30 (24 females) patients were recruited to the study; however, 5 recruits (5 females) failed to complete all of the tests and 1 male withdrew due to a previously undiagnosed cardiac complaint (Figure 1). The demographic data, disease characteristics, and disability scores of the remaining 24 patients are given in Table 1.

The step test was well tolerated, with no adverse events. All of the 24 patients completed both step tests in a single stage. Two female patients did not complete the graded exercise test for the determination of $\overset{.}{\text{V}}{\text{O}}_{2\;\text{peak}}$. Mean peak values for HR, $\overset{.}{\text{V}}{\text{O}}_{2}$, RER, and RPE of the remaining 22 patients are presented in Table 2.

### 3.1. Validity Analyses

**Figure 2:**
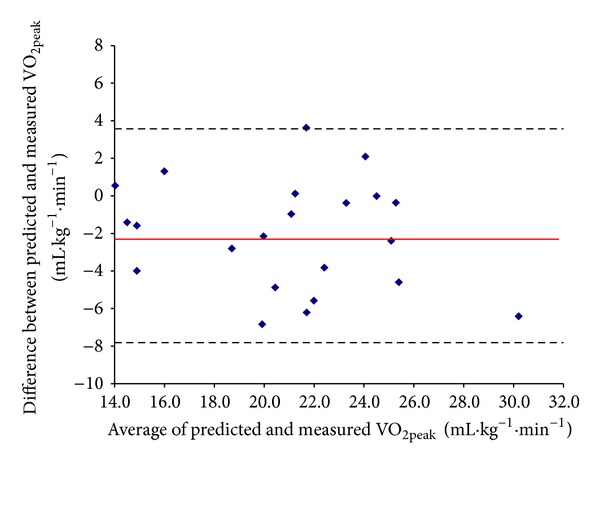
Bland-Altman plot of $\overset{.}{\text{V}}{\text{O}}_{2\;\text{peak}}$ measured during the cycle test and $\overset{.}{\text{V}}{\text{O}}_{2\max}$ predicted by the Siconolfi step test (visit 2). The mean bias is represented by the solid line and the 95% limits of agreement are represented by the dashed lines.

The mean values for $\overset{.}{\text{V}}{\text{O}}_{2\max}$ estimated from the second step test (Visit 2) and directly measured $\overset{.}{\text{V}}{\text{O}}_{2\;\text{peak}}$ were (22.0 ± 4.5) and (19.9 ± 4.2) mL·kg^−1^·min^−1^, respectively (*P* = 0.003). The Pearson correlation coefficient (*r*) was 0.79 (95% CI 0.55 to 0.91). When $\overset{.}{\text{V}}{\text{O}}_{2\max}$ estimated from the first step test (Visit 1) was used for the same analyses, the findings were found to be very similar (*r* = 0.77; 95% CI 0.52 to 0.90). The Bland-Altman plot of within-subject differences between estimated $\overset{.}{\text{V}}{\text{O}}_{2\max}$ and directly measured $\overset{.}{\text{V}}{\text{O}}_{2\;\text{peak}}$ versus the mean of the two tests is presented in Figure 2. The systematic bias between estimated $\overset{.}{\text{V}}{\text{O}}_{2\max}$ and directly measured $\overset{.}{\text{V}}{\text{O}}_{2\;\text{peak}}$ was 2.1 mL·kg^−1^·min^−1^, the 95% limits of agreement (LoA) were ±5.7 mL·kg^−1^·min^−1^, and the SEE was 2.6 mL·kg^−1^·min^−1^ (95% CI 2.0 to 3.8 mL·kg^−1^·min^−1^).

### 3.2. Reliability Analyses

**Figure 3:**
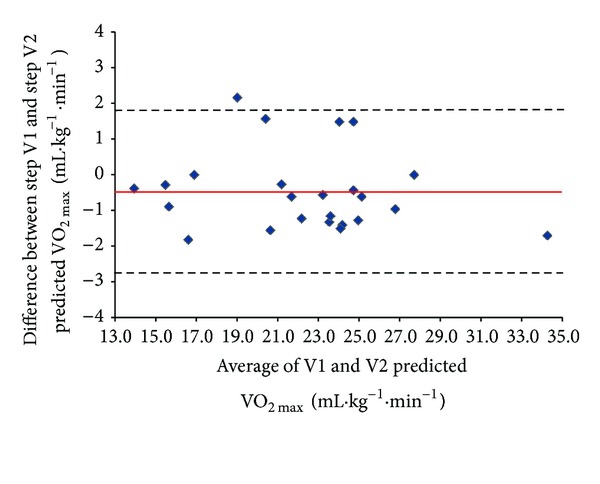
Bland-Altman plot of $\overset{.}{\text{V}}{\text{O}}_{2\max}$ predicted by the Siconolfi step test on visit 1 and visit 2. The mean bias is represented by the solid line and the 95% limits of agreement are represented by the dashed lines.

Data for sub-maximal step tests are summarized in Table 3. The mean value for $\overset{.}{\text{V}}{\text{O}}_{2\max}$ estimated at Visit 1 (22.5 ± 4.7 mL·kg^−1^·min^−1^) was marginally, albeit significantly, higher than that at Visit 2 (22.0 ± 4.5 mL·kg^−1^·min^−1^,  *P* = 0.049). The intraclass correlation coefficient (ICC) was 0.97 (95% CI of 0.94 to 0.99), and the Pearson correlation coefficient (*r*) was 0.97 (95% CI 0.93 to 0.99). The Bland-Altman plot (Figure 3) of the within-subject change for estimated $\overset{.}{\text{V}}{\text{O}}_{2\max}$ versus the mean for both step tests (i.e., Visit 1 and Visit 2) indicates a small systematic bias (−0.5 mL·kg^−1^·min^−1^) between the first and second tests. The 95% limits of agreement (LoA) were ±2.2 mL·kg^−1^·min^−1^. The within-subject coefficient of variation for estimated $\overset{.}{\text{V}}{\text{O}}_{2\max}$ was 5.4%.

## 4. Discussion

The findings presented here demonstrate that administration of the Siconolfi step test provides a valid and reproducible estimation of cardio-respiratory fitness ($\overset{.}{\text{V}}{\text{O}}_{2\max}$) in routine clinical practice. These findings are important; $\overset{.}{\text{V}}{\text{O}}_{2\max}$, the measure of an individual's cardio-respiratory fitness, is a strong independent predictor of mortality in asymptomatic individuals as well as in clinical patients. Low cardio-respiratory fitness carries the same or higher strength of association or risk for mortality as routinely measured clinical risk factors such as hypertension, hyperlipidemia, diabetes, family history of CVD, and smoking [10]. Furthermore, meta-analysis indicates that a cardio-respiratory fitness below ~28 mL·kg^−1^·min^−1^ results in substantially higher rates of all-cause mortality and CHD/CVD events in healthy persons [31]. This is alarming considering the fact that the average cardio-respiratory fitness level of the RA patients in this investigation was  19.9 ± 4.2 mL·kg^−1^·min^−1^. Despite all the evidence to support the use of cardio-respiratory fitness as an additional clinical measure for identifying CVD risk, assessment of $\overset{.}{\text{V}}{\text{O}}_{2\max}$ is usually not performed in most, if not all, clinical practices.

Direct measurement of $\overset{.}{\text{V}}{\text{O}}_{2\max}$ may place certain patient groups like RA patients at risk and is not always practical in many healthcare settings. In contrast, estimation of $\overset{.}{\text{V}}{\text{O}}_{2\max}$ from sub-maximal testing appears to have greater applicability, particularly for assessment of cardio-respiratory fitness in a clinical setting. Sub-maximal predictive tests like the Siconolfi step test provide a simple, safe, and valid estimate of $\overset{.}{\text{V}}{\text{O}}_{2\max}$. Originally developed to estimate $\overset{.}{\text{V}}{\text{O}}_{2\max}$ in apparently healthy individuals, the purpose of the present study was to determine if administration of the Siconolfi step test provided a valid and reliable estimate of $\overset{.}{\text{V}}{\text{O}}_{2\max}$ in patients with RA, a population with increased CVD risk and low exercise tolerance.

The findings presented here indicate that the Siconolfi step test is a valid measure of cardio-respiratory fitness in patients with RA. $\overset{.}{\text{V}}{\text{O}}_{2\max}$ estimation from the Siconolfi step test was strongly correlated with measured $\overset{.}{\text{V}}{\text{O}}_{2\;\text{peak}}$ (*r* = 0.79). $\overset{.}{\text{V}}{\text{O}}_{2\max}$ estimation from the Siconolfi step test was also in reasonable agreement with the criterion measure, that is, directly measured $\overset{.}{\text{V}}{\text{O}}_{2\;\text{peak}}$. However, there was a small significant positive bias in the estimated versus the actual $\overset{.}{\text{V}}{\text{O}}_{2\max}$. The bias indicated that the Siconolfi step test could potentially overestimate $\overset{.}{\text{V}}{\text{O}}_{2\max}$ by 3.6 mL·kg^−1^·min^−1^ in RA patients. Furthermore, the overall standard error of estimate means that the accuracy of the $\overset{.}{\text{V}}{\text{O}}_{2\max}$ estimation in RA patients with an actual $\overset{.}{\text{V}}{\text{O}}_{2\max}$ ranging from 12.9 to 27.0 mL·kg^−1^·min^−1^ was approximately 10 to 20%.

An estimated value for $\overset{.}{\text{V}}{\text{O}}_{2\max}$ that is higher than the directly measured $\overset{.}{\text{V}}{\text{O}}_{2\;\text{peak}}$ may reflect differences between stepping and cycling exercise. It is possible that local muscle fatigue experienced by those unaccustomed to cycling exercise may have resulted in some of the maximal exercise tests being terminated before attainment of “true” $\overset{.}{\text{V}}{\text{O}}_{2\max}$. However, other indicators of maximal effort concomitant with $\overset{.}{\text{V}}{\text{O}}_{2\max}$ [28], such as attaining a heart rate within 15 bpm of the age-predicted maximal value, a respiratory exchange ratio of greater than 1.10, and RPE greater than 17, were achieved in RA patients. This suggests that the current patients did exercise at or close to their maximal effort. Another possible explanation for overestimation of cardio-respiratory fitness by the step test may be related to the timing of tests performed on visit 2. However, the short duration of the step test (i.e., 3 minutes) and the longer rest period (i.e., 30 minutes minimum) between tests argue against this.

The test-retest repeatability of the estimated $\overset{.}{\text{V}}{\text{O}}_{2\max}$ via the step test in the current study was excellent. The Pearson correlation coefficient and ICC indicated a very strong positive correlation between the two step tests. Thus, we conclude that the Siconolfi step test is a reliable measure of cardio-respiratory fitness in patients with RA. There was a small but significant intertrial bias (−0.5 mL·kg^−1^·min^−1^). However, the 95% LoA (±2.2 mL·kg^−1^·min^−1^) is considered acceptable. Thus, an increase in estimated $\overset{.}{\text{V}}{\text{O}}_{2\max}$ of approximately 2.5 mL·kg^−1^·min^−1^ or above following an exercise training intervention could be considered a change that is due to factors other than chance. In RA patients, this would equate to an increase in cardio-respiratory fitness of around 20% for the individual with the lowest $\overset{.}{\text{V}}{\text{O}}_{2\max}$, whereas the person with the highest $\overset{.}{\text{V}}{\text{O}}_{2\max}$ would experience a 10% increase.

We know of only one other study that has investigated the validity and test-retest reproducibility of the Siconolfi step test in a patient group. Marcora and colleagues [32] found that the Siconolfi step test was reasonably valid and highly reliable in patients with well-controlled systemic lupus erythematosus (SLE). Compared to the patients in the present study, the SLE patients in that study were younger, weighed slightly less, had similar BMI, and had a higher directly measured $\overset{.}{\text{V}}{\text{O}}_{2\max}$ relative to body mass. The validity and reliability analyses for our study compare well with those of Marcora et al.

The most concerning, but unsurprising finding, of this study is the very low value for the directly measured $\overset{.}{\text{V}}{\text{O}}_{2\max}$ in our RA patients. Previous reports indicate that $\overset{.}{\text{V}}{\text{O}}_{2\max}$ may be 20 to 30% lower in RA patients compared with age-matched healthy controls [33–36]. A major determinant of $\overset{.}{\text{V}}{\text{O}}_{2\max}$ is the degree of physical activity over recent weeks and months. Evidence suggests that approximately 68% of RA patients in the UK are physically inactive [37]; therefore, compromised cardio-respiratory fitness in patients with RA is hardly surprising. Despite knowing that increased incidence of CVD-related morbidity and mortality is a common feature of RA [38, 39], the relative contributions of physical inactivity, traditional risk factors, and high grade systemic inflammation to the exacerbated CVD risk in this population remain unclear [40–43]. However, exercise is recognized as useful adjunct treatment for RA [44], although the relationships between cardio-respiratory fitness, exercise training, and CV risk in RA patients require more research [43].

The strengths and weaknesses of this study warrant comment. The Siconolfi step test is easy to administer, requires minimal equipment, and is relatively quick since it can be completed at low levels of exercise. Thus, there is considerable potential for its use as a clinical tool for routine assessment of cardio-respiratory fitness in patients with RA and other clinical populations who are at risk of developing CVD. All of the patients studied here completed the test after the first stage. Although fatigue, pain, limited joint mobility, and impaired muscle strength are all common features of RA [22, 35], the step test was reasonably well tolerated by patients in this study. Potential sources of error in the study include prediction of maximum heart rate from the 220—age formula, assumption of a linear relationship between heart rate and $\overset{.}{\text{V}}{\text{O}}_{2}$, and the individual's ability to maintain the correct stepping tempo, all of which are common to sub-maximal exercise testing [28]. Due to RA being primarily a joint disease, we specifically chose to compare the step test to a cycling-based $\overset{.}{\text{V}}{\text{O}}_{2\max}$ test. Even though cycling is not a weight-bearing activity, it may put the knee joint under a similar strain/range of movement than a walking-based test. A treadmill-based $\overset{.}{\text{V}}{\text{O}}_{2\max}$ test may have resulted in a higher $\overset{.}{\text{V}}{\text{O}}_{2\max}$ than what was obtained in this study [17]; however, our $\overset{.}{\text{V}}{\text{O}}_{2\max}$ results compare well to other studies that also used sub-maximal cycle tests to estimate RA patient fitness levels [45]. Another limitation is the modest sample size; however, it was sufficient to meet the study objectives, with suitable measures of validity and test-retest reliability being observed. Finally, the suitability of the step test as a measure of cardio-respiratory fitness from a clinical perspective warrants comment. The average discrepancy between predicted and actual $\overset{.}{\text{V}}{\text{O}}_{2\max}$ was 2.1 mL·kg^−1^·min^−1^. In general this discrepancy is acceptable and expected of a predictive sub-maximal exercise test like the Siconolfi step test. However, when interpreting the estimated values provided by the step test, it must be noted that there is a trend for the discrepancy to increase when average cardio-respiratory fitness levels are lower.

## 5. Conclusions

The present study is the first to demonstrate that the Siconolfi step test is a valid and reliable method for assessing cardio-respiratory fitness in an RA population. In light of considerable epidemiological evidence that supports the cardioprotective effects of regular physical activity and cardio-respiratory fitness, the current findings indicate a role for simple, clinically available physiological estimation of $\overset{.}{\text{V}}{\text{O}}_{2\max}$. Another important finding is the very low cardio-respiratory fitness in patients with RA when assessed using both step and cycle tests. It is well known that this group is twice as likely to die from a CVD-related event when compared to the general population [39]. Therefore, following on from the current study it is believed that patients with RA and other chronic diseases with increased risk of CVD should have their cardio-respiratory fitness measured as part of their cardiovascular screening and are advised to maximise as part of any long-term management plan. This is achievable using the step test in RA.

## Figures and Tables

**Table 1:** Characteristics of 24 patients (19 females and 5 males) with RA participating in the study.

	Females	Males	Total group
Age (years)	54.5 ± 10.5	49.4 ± 9.5	53.4 ± 10.4
Weight (kg)	69.5 ± 15.3	90.0 ± 17.0	73.8 ± 17.5
Height (cm)	164.0 ± 5.6	178.6 ± 6.3	167.1 ± 8.6
BMI (kg·m^−2^)	25.8 ± 5.1	28.3 ± 5.1	26.3 ± 5.1
Resting SBP (mmHg)	125.0 ± 2.0	139.0 ± 7.0	128.0 ± 11.0
Resting DBP (mmHg)	79.0 ± 1.0	86.0 ± 2.0	81.0 ± 6.0
Disease duration (years)	13.0 ± 1.9	12.8 ± 1.8	13.0 ± 7.3
DAS 28 ESR	2.9 ± 0.3	2.0 ± 0.3	2.7 ± 1.1
HAQ (0–3)	0.6 (range 0 to 1.6)	0.2 (range 0 to 0.4)	0.5 (range 0 to 1.6)

Values are mean ± SD or range. BMI: body mass index; SBP: systolic blood pressure; DBP: diastolic blood pressure; DAS: disease activity score; ESR: erythrocyte sedimentation rate, HAQ: health assessment questionnaire.

**Table 2:** Physiological variables from the maximal cycling ergometry test in 22 patients (17 females and 2 males).

	Females	Males	Total group
HR_peak_	158 ± 13	159 ± 22	158 ± 15
Age predicted max (%)	95 ± 6	93 ± 8	95 ± 7
${{\dot{\text{V}}\text{O}}_{\text{2}}}_{\text{ peak}}$ (mL·kg^−1^·min^−1^)	19.2 ± 4.1	22.3 ± 4.0	19.9 ± 4.2
RER_peak_ ($\dot{\text{V}}$O_2_/$\dot{\text{V}}$CO_2_)	1.18 ± 0.12	1.14 ± 0.04	1.18 ± 0.11
RPE (15 point scale)	19 ± 2	18 ± 2	19 ± 2

Values are mean ± SD. RER: respiratory exchange ratio; RPE: ratings of perceived exertion; $\dot{\text{V}}$O_2_: oxygen consumption; $\dot{\text{V}}$CO_2_: carbon dioxide production.

**Table 3:** Heart rate (bpm and % age predicted maximum) and corresponding estimated $\overset{.}{\text{V}}{\text{O}}_{2\max}$ for 24 patients (19 females and 5 males) that performed the Siconolfi step test.

	Visit 1			Visit 2		
	HR	% Age predicted max	Estimated $\overset{.}{\text{V}}{\text{O}}_{2\max}$	HR	% Age predicted max	Estimated $\overset{.}{\text{V}}{\text{O}}_{2\max}$
Females	122 ± 12	74 ± 9	21.2 ± 0.9	125 ± 14	75 ± 9	20.8 ± 0.9
Males	120 ± 10	70 ± 4	27.6 ± 1.9	127 ± 14	74 ± 6	26.7 ± 1.8

Total group	122 ± 11	73 ± 8	22.5 ± 4.7	125 ± 14	75 ± 8	22.0 ± 4.5

Values are mean ± SD. $\dot{\text{V}}$O_2_: oxygen consumption; HR: heart rate, BPM: beats per minute.
